# Vascular Labeling of Extracranial Head and Neck Vessels Using Silicone Dye Injection: An Effective Method for Cadaver-Based Facial Surgery Training

**DOI:** 10.1055/s-0044-1792154

**Published:** 2024-11-08

**Authors:** Raja Tiwari, Renu Dhingra, Sanjeev Lalwani, Maneesh Singhal

**Affiliations:** 1 Department of Plastic, Reconstructive and Burns Surgery, All India Institute of Medical Sciences, New Delhi, India; 2 Department of Anatomy, All India Institute of Medical Sciences, New Delhi, India; 3 Division of Forensic Pathology and Molecular DNA Laboratory, All India Institute of Medical Sciences, New Delhi, India

**Keywords:** silicone dye, vascular labeling, dye study, head and neck, dye injection

## Abstract

**Background** Silicone dye injection is a well-described technique for studying vascular anatomy. Plastic surgeons routinely participate in cadaveric workshops and are involved in the preparation of vascular-labeled cadavers. However, sparse literature is available on dye studies of extracranial head and neck vessels, even with anatomists, and the preparation of these cadavers is a daunting task.

**Materials and Methods** In this study, we describe a straightforward technique of silicone dye injection for extracranial head and neck vasculature and its application for filler injection training and other plastic surgical procedure demonstrations on cadavers. We used six soft-embalmed cadavers. The common carotid arteries and internal jugular veins were cannulated with infant feeding tubes. The vessels that could lead to dye extravasation into intracranial vessels and upper limbs were ligated. The vasculature was irrigated with water and then injected with color-coded silicone dyes (red: arterial; blue: venous). The solvent-to-catalyst ratios were varied to identify the ideal combination. Injected specimens were dissected 24 hours later to identify the staining quality.

**Results** A clear demarcation of the extracranial head and neck vasculature in all cadavers was seen. The best solvent-to-catalyst ratio was identified in cadavers with the best staining of both large and small-sized blood vessels (<1 mm) with no dye spillage from arteries to the veins.

**Conclusion** Silicone dye injection with the described technique can give excellent and predictable results. The technique also uses less quantity of dye, and the intracranial structures are spared, which can be used for other studies; hence, there is more economical utilization of cadavers.

## Introduction

Labeling cadaver vasculature using colored dyes is a valuable technique for anatomical studies of various body parts. Selective dye staining of the extracranial vessels preserves the intracranial vasculature for other uses, allowing economical cadaveric use. Selective silicon dye staining of the extracranial vessels highlights all facial blood vessels, thus enabling excellent demonstrations of filler injection and other plastic surgical procedures.

While several studies have explored techniques for staining specific extracranial head and neck regions, such as the maxillary sinus and perioral area, these methods have notable limitations. 1 2 Another study described the use of latex dyes for staining extracranial vessels, but the process was cumbersome. The latex dye often fails to enter smaller vessels due to its viscosity and could cause swelling or gas production due to latex allergy. 3 In contrast, silicone dyes are well documented in the literature and are effectively used for vascular studies in other body parts.

Understanding facial vascular anatomy is crucial for procedures like filler injections to prevent complications such as bruising, swelling, necrosis, pigmentation, or infection. 4 This study presents a technique for selective silicone dye labeling of the extracranial head and neck vasculature, with color-coded arterial and venous staining. We also outline the standard solvent and catalyst concentrations and polymerization times to effectively color the smallest blood vessels and prevent any arterial dye pooling into the venous circulation along with addressing possible complications in vascular labeling and methods to avoid them. Additionally, we discuss a cadaver-based facial anatomy training technique, specifically for filler injections, aimed at minimizing the complications associated with it.

## Materials and Methods

This prospective observational study was conducted at All India Institute of Medical Sciences (AIIMS), New Delhi. The methodology used is discussed in the following sections.

### Acquisition and Preparation of Cadavers

Ethical approval was obtained from the institutional ethical committee following the World Medical Association Declaration of Helsinki. Six soft-embalmed cadavers, donated to our anatomy department, were used for head and neck vasculature study using silicone dye injection. Embalming was done as per the method described by Thiel. 5 All the cadavers had been prepared following the regulations of donations. Decapitation was avoided to maintain cadaver reusability.

### Dissection, Cannulation, and Dye Injection

**Fig. 1:**
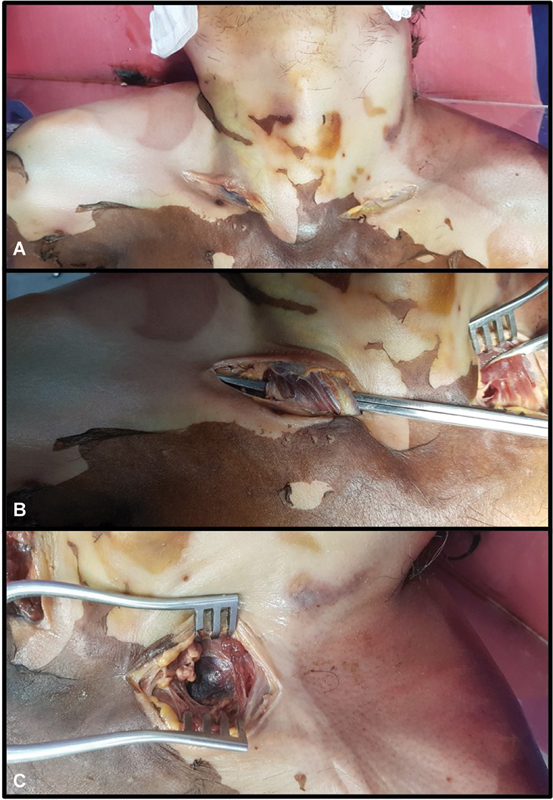
( **A** ) Skin incisions made in the supraclavicular area. ( **B** ) Sternocleidomastoid muscle identified and dissected. ( **C** ) Carotid sheath visualized.

**Fig. 2:**
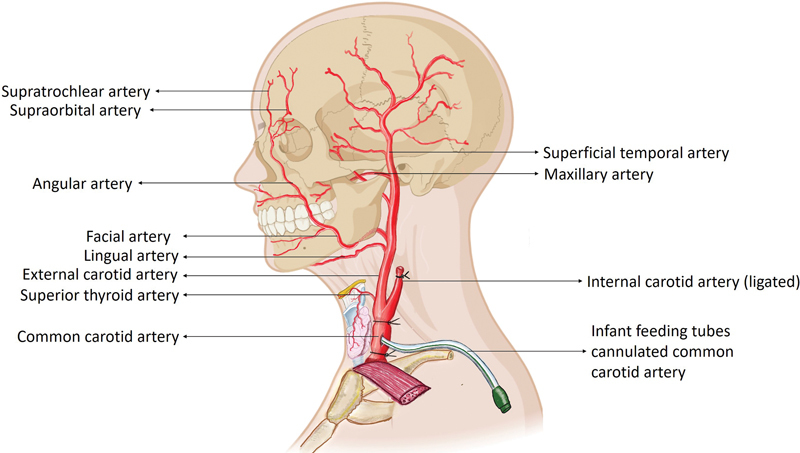
Extracranial arterial anatomy of the head and neck. Note the feeding tube cannulated into the common carotid artery and the ligated internal carotid artery.

**Fig. 3:**
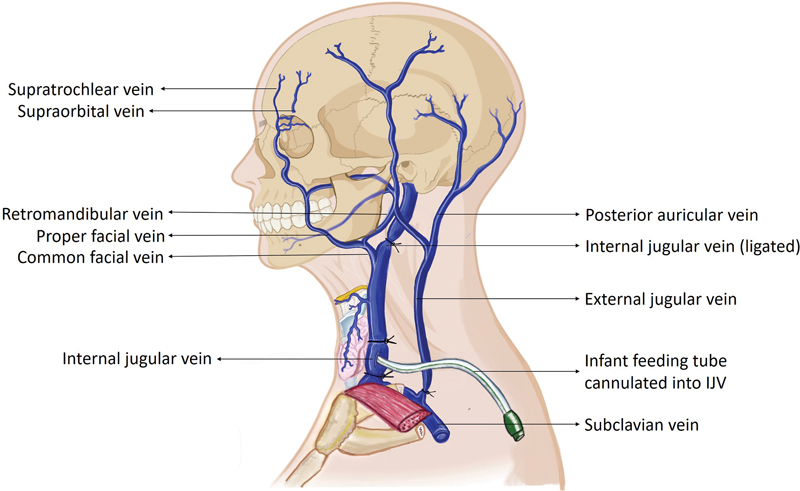
Extracranial venous anatomy of the head and neck. Note the feeding tube cannulated into the internal jugular vein and the ligated internal jugular vein distal to the origin of the common facial vein.

An incision was made over the supraclavicular area ( Fig. 1a ). After elevating the skin flaps, the sternocleidomastoid muscle was divided to expose the carotid sheath ( Fig. 1b, c ). The internal carotid artery (ICA) and subclavian artery (SCA) were then ligated near their origin ( Fig. 2 ). Following this, the internal jugular vein (IJV) was ligated distal to the origin of the common facial vein (CFV), while the external jugular vein was ligated close to its entrance into the subclavian vein (SCV; Fig. 3 ). The same procedure was performed on the contralateral side. The dissection was meticulously done, and branches of the carotid arteries or tributaries of the jugular vein were preserved. The small vessels that got inadvertently injured were ligated.

The common carotid arteries and IJVs were cannulated using infant feeding tubes (14 Fr), which were secured with ligatures to prevent dye leakage from the entry site. The vessels were then ligated proximal to the cannulation site to prevent any backflow toward the heart and other systems, ensuring the cadaver's reusability ( Figs. 2 and 3 ). The arterial system was irrigated with saline using a 50-mL syringe, and the returning fluid was allowed to drain through the cannulated IJV on the same side until it became clear. Similar irrigation was then performed through the IJV and allowed to drain through the carotid artery. The same procedure was also performed on the contralateral side. During this process, the face typically begins to appear swollen due to fluid extravasation.

**Fig. 4:**
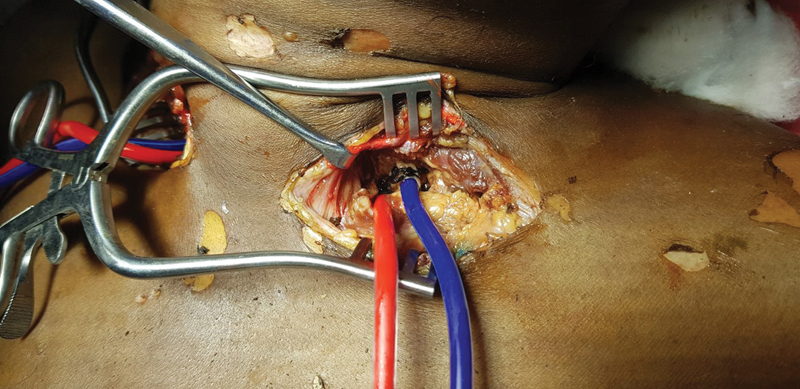
Color-coded silicone dyes ( *red and blue* ) injected into the arterial and venous systems, respectively.

Blue and red colored silicone rubber dyes (Biodur GmBh, Germany) were prepared using solvent (S10), colors (AC50 for red and AC52 for blue), and a catalyst (S1) for polymerization. The viscosity and polymerization time of the dyes were adjusted by varying the proportions of solvent and catalyst, as described in Table 1 . The color-coded silicone dyes (red: arterial; blue: venous) were then injected slowly through the feeding tubes using 50-mL syringes, applying sufficient pressure to allow the dyes to move gradually into the external carotid artery (ECA) ( Fig. 4 ). As the dye began to polymerize, resistance to injection increased, and a point was reached where no additional dye could be injected. The red dye can be seen staining the oral and labial mucosa, and the skin showed a pinkish hue. The adequacy of color injection was verified over the area of parietal eminence as it represents the most distal site of blood flow from the ECA. The ends of the feeding tubes were then clamped. The blue dye was subsequently injected into the venous system in a similar manner until no more dye could be added, even with full manual pressure. The total volume of dye used in the arterial system was around 74 ± 11 mL on each side. The cadavers were then stored for 24 hours before the demonstration to allow excess fluid to dissipate and facial swelling to subside.

**Table 1:** Composition and polymerization times of dyes used in different cadavers

Groups (total volume: 50 mL)	Solvent (S10) volume (mL)	Colors (AC50 for red and AC52 for blue dye), mL	Catalyst (S1) volume (mL)	Polymerization time (min)
Cadavers 1 and 2	37.5	2.5	10	5
Cadavers 3 and 4	40	2.5	7.5	15
Cadavers 5 and 6	42.5	2.5	5	30

### Dissection of Extracranial Head and Neck Vasculature

**Fig. 5:**
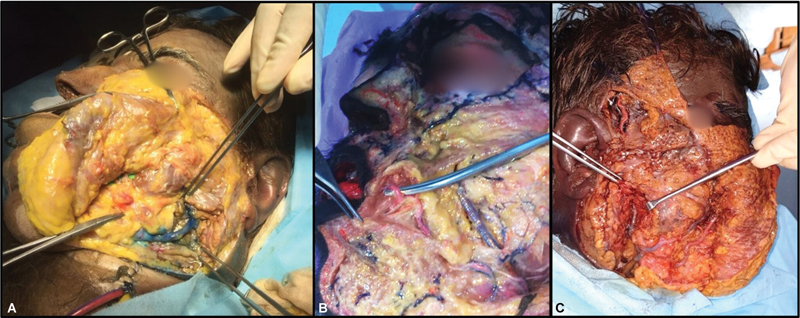
Varying quality of facial vessels staining. ( **A** ) Good staining in larger vessels with poor staining in smaller vessels. ( **B** ) Good staining in both large and small vessels. ( **C** ) Good staining in both, but with dye overflow into the venous systems.

Twenty-four hours after the dye injection, one side of the face was first dissected to demonstrate the facial tissue layers and the vascular anatomy. The dissection involved the sequential elevation of various layers of the head and neck (skin, subcutaneous tissue, superficial musculoaponeurotic system, and facial musculature). The quality of color-coded vascular labeling was noted for each cadaver ( Fig. 5 ).

### Demonstration of Filler Injection Technique

**Fig. 6:**
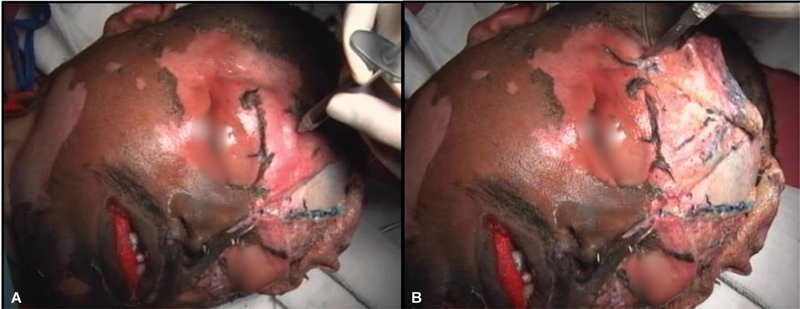
( **A** ) Filler injection on the right forehead. ( **B** ) Layer-wise dissection demonstrates vascular anatomy and correct filler placement.

After understanding the facial vasculature on one side; the usefulness of vascular labeling was demonstrated by injecting colored fillers into the contralateral side of the face. These fillers were injected after identifying the surface anatomy of the blood vessels in the respective zones, thus safeguarding them ( Fig. 6a ). Following the filler injection, this side of the face was dissected layer by layer ( Fig. 6b ). During the dissection, any vessel damage resulting from the colored filler injection was carefully noted.

## Results

There was a clear demarcation of extracranial head and neck vasculature in all the cadavers, with arterial and venous systems distinctly identifiable. However, variations in vascular staining were observed among different cadavers. In cadavers 1 and 2, the shorter polymerization time of the dyes highlighted larger vessels, such as the facial vessels, but missed smaller vessels ( Fig. 5a ). In contrast, cadavers 3 and 4, which had an intermediate polymerization time, resulted in good visualization of both large and small vessels, including those smaller than 1 mm in diameter ( Fig. 5b ). Cadavers 5 and 6 exhibited good dye penetration into both large and small vessels; however, there was some overflow of dye from the arterial into the venous system ( Fig. 5c ). These observations are summarized in Table 2 .

**Table 2:** Characteristics of dye staining in different cadavers

Dissection findings	Cadavers 1 and 2	Cadavers 3 and 4	Cadavers 5 and 6
Visualization of large arteries	Good	Good	Good
Visualization of small arteries	Poor	Good	Good
Pooling of red dye into venous system	Nil	Nil	Yes

On the contralateral side, the vascular anatomy closely mirrored that of the side initially dissected. The filler injections after demonstration of the vascular anatomy were all found to be within safe zones of the face, with no instance of inadvertent intra- or perivascular injection.

## Discussion

Detailed knowledge of head and neck vasculature is crucial for better surgical planning and avoiding inadvertent complications. This study demonstrates the usefulness of dye staining of the extracranial head and neck vessels for training in filler injections, a popular nonsurgical cosmetic procedure. A proper understanding of the anatomy, injection techniques, and filler properties is essential. Using simulated procedures on cadavers is an effective method for teaching both anatomical concepts and proper injection techniques.

Studies involving decapitated cadavers, while useful, have several limitations. 6 The decapitation, typically performed at the fourth or fifth cervical vertebra, removes the cricoid cartilage, 6 thus affecting the studies involving the neck. Additionally, the raw surface at the cut end often leads to significant dye leakage through small vessels. Therefore, using a nondecapitated head offers a more economical use of dye and preserves the cadaver for future studies.

While vascular labeling of intracranial head and neck anatomy is well documented, 7 8 there is limited literature on extracranial vessels. Some studies have focused on specific regions, such as the maxillary sinus and perioral area, using colored latex dyes. 1 2 However, these studies did not ligate the ICA, flush vessels before dye injection, or assess the quality of staining.

Injection techniques for labeling vascular anatomy have been used for centuries, starting with Jean Riolan in the 17th century. Over time, various dyes have been introduced, with latex being the most commonly used. However, latex presented several challenges: its viscosity often prevented it from entering smaller vessels and it posed risks of swelling or gas production due to potential allergy reactions. 3 Also, the presence of liquid in the vessels can affect its proper diffusion; hence, drying of the vessel lumen becomes a critical step before injection. In contrast, silicone dyes offer several advantages. They do not require vessels to be dried before injection, as their viscosity effectively expels any remaining fluid, avoiding the complications associated with latex dyes.

We selected silicone dyes in our study due to their ease of preparation, handling, and favorable setting time. These dyes not only effectively stain the blood vessels but also provide reliable structural support after polymerization, preserving the three-dimensional vascular anatomy and facilitating dissection. Moreover, this technique allows for easy identification of vessels smaller than 1 mm, making it valuable for other research applications like flap harvesting, perforator localization, and studying anatomical variations.

Our experience with color-coded silicone dyes for vascular labeling of extracranial head and neck has allowed us to standardize the technique and reduce dye usage. We achieved satisfactory dye staining of the supratrochlear, supraorbital, and dorsal nasal arteries (branches of internal carotid system) through their anastomotic communications with the angular artery. For optimal results, we recommend a dye preparation with 40 mL of solvent (S10) mixed with 7.5 mL of catalyst (S1) and a polymerization time of 15 minutes. This mixture ensures better dye penetration into smaller-caliber vessels without any venous spillover. A crucial aspect of the irrigation and dye injection process is the application of adequate pressure. An adequate pressure ensures full vascular perfusion without damaging the vessels. 9 We used 50-mL syringes for both irrigation and dye injection, monitoring the syringe plunger tension carefully to gauge resistance and avoid vascular damage.

We utilized filler injections to demonstrate the usefulness of our vascular labeling technique as vascular complications such as hematomas, occlusions, and ischemic necrosis 4 10 11 12 13 are common after filler injection. Vascular occlusion can occur in up to 3 in 1,000 cases, 10 11 particularly in the glabellar region. 13 Smaller vessels are more prone to occlusion from perivascular injections, while larger vessels can be blocked by either peri- or intravascular injections, leading to localized ischemia or extensive tissue loss. 4 Severe complications including blindness, 14 15 pulmonary embolization, 16 and stroke 13 can occur due to embolization of intravascular filler injection.

Based on the understanding of the vascular anatomy of the face, facial danger zones have been described, which guide the surgeon in injecting the fillers safely. 17 In the upper face, knowledge of superficial temporal, supraorbital, supratrochlear, and dorsal nasal arteries is crucial. 18 19 In the lower face, the facial, labial, angular, infraorbital, and mental arteries are important. 19 20 Detailed vascular anatomy can be better learned through simulated training on vascular-labeled cadavers.

Our vascular labeling technique with silicone dyes achieved good penetration in arteries smaller than 1 mm. It was found that the viscosity of the dye following 15 minutes of polymerization was satisfactory for studying small vessels. The arteries in facial danger zones, which are crucial for safe filler injection, 17 were easily identified during our dissection, enhancing trainees' understanding of the complex extracranial vascular network. Thus, demonstration of vascular anatomy on labeled cadavers can help amateur injectors better understand the facial anatomy and danger zones, where proficiency in vascular anatomy can prevent complications.

This study has limitations of its own; if one hemiface is already dissected, demonstrating injection techniques on the other side may reduce the risk of injury due to improved anatomical understanding. Additionally, the small sample size calls for future studies with larger samples to confirm our findings and recommendations.

## Conclusion

Silicone dye injection using our technique yields reliable and consistent results while maximizing the efficient use of dye and cadavers. A standardized vascular labeling method, with a 15-minute polymerization time (40 mL of solvent [S10] mixed with 7.5 mL of catalyst [S1]), offers the best outcomes. Training on vascular-labeled cadavers serves as an excellent technique to educate practitioners about the danger zones in filler injections and to prepare cadavers for hands-on filler injection programs.
